# Long-Term Outcomes of Chronic Hemodialysis Patients Following SARS-CoV-2 Infection

**DOI:** 10.7759/cureus.92535

**Published:** 2025-09-17

**Authors:** Asmaa Bouchari, Fouad Dami, Mariam El Hammouti, Soukaina Ben Driss Alami, houda Hanafi, Sara El Maakoul, Maryam Assem

**Affiliations:** 1 Nephrology Department, Mohammed VI University Hospital, Tangier, MAR

**Keywords:** chronic hemodialysis, covid-19, long covid, risk factors, symptoms

## Abstract

Introduction: The COVID-19 pandemic has had a significant impact on patients undergoing chronic hemodialysis (CHD). Several studies have explored the long-term outcomes of CHD patients who recovered from SARS-CoV-2 infection. This study aims to identify the factors associated with persistent symptoms (long COVID) among CHD patients.

Methods: We conducted a multicenter, descriptive, and analytical cohort study across nine hemodialysis centers (public, private, and nonprofit) in the city of Tangier. Data were collected through interviews with patients and a review of their medical records. Statistical analysis was performed using IBM SPSS Statistics version 25.

Results: Among 945 CHD patients, 163 had a documented SARS-CoV-2 infection, with a median age of 55 years (interquartile range (IQR): 43-67). The most frequently reported post-infection symptoms were fatigue (62%), anxiety (53%), arthralgia (40%), cough (33%), weight loss (29%), sleep perturbations (28%), anosmia (30%), dyspnea (25%), anorexia (24%), dysgeusia (18%), and concentration difficulties (16%). After adjusting for diabetes, obesity, oxygen therapy, hospitalization, and use of hydroxychloroquine or corticosteroids, diabetes emerged as the main factor for long COVID (OR = 3.8; 95% CI (1.5-9.6); p = 0.004). Fatigue was significantly associated with diabetes (OR = 2.9; 95% CI (1.3-6.5); p = 0.01) and female gender (OR = 2.1; 95% CI (1.02-4.2); p = 0.04). Anxiety was linked to diabetes (OR = 2.6; 95% CI (1.2-5.5); p = 0.01) and obesity (OR = 2.5; 95% CI (1.01-6.4); p = 0.04). Dyspnea was associated with obesity (OR = 4.0; 95% CI (1.4-10.4); p = 0.004).

Conclusion: Our study highlights the substantial prevalence of persistent post-COVID-19 symptoms among CHD patients. The findings emphasize the necessity of individualized long-term care in this high-risk group.

## Introduction

The COVID-19 pandemic, caused by the SARS-CoV-2 virus, has resulted in an unprecedented global health crisis due to both its rapid transmission and the severity of its complications. While the initial clinical manifestation primarily involves the respiratory system, often as pneumonia that can progress to acute respiratory distress syndrome (ARDS), beyond that, COVID-19 can affect multiple organs, notably the heart, kidneys, liver, and nervous system, and may result in thromboembolic complications [1].

Maintenance hemodialysis (MHD) patients are particularly vulnerable to severe outcomes. This heightened risk is largely attributed to a high prevalence of comorbidities such as hypertension, diabetes, and cardiovascular diseases, compounded by the immunocompromised state associated with end-stage renal disease (ESRD) and the seniors of this population [2]. Multiple studies showed that these factors contribute to higher mortality and prolonged morbidity in MHD patients infected with SARS-CoV-2 [3,4].

While many studies have explored the acute phase of the infection, the medium- and long-term consequences, particularly the persistence or recurrence of symptoms, referred to as “long COVID," remain insufficiently documented in this specific population [5].

In this context, the objective of our study is to assess the prevalence of long COVID and to identify the factors associated with the persistence of post-COVID-19 symptoms in MHD patients. This investigation aims to enhance the understanding of long-term outcomes by detailing the impact on quality of life and to contribute to improving overall management by developing specific strategies for this high-risk group.

## Materials and methods

Study design

We conducted a multicenter retrospective study over a 36-month period, from January 2020 to December 2022, involving nine hemodialysis centers (public, private, and nonprofit) in the city of Tangier, Morocco: Mohammed V Hospital in Tangier (a public facility), Alboughaz Center 40, Ibn Rochd Center 25, Tangier Center, Al Hayat Center, Avicenne Center, ENNOUR Center, AMAL Association, and Souriyenne Center). The study included MHD patients with a confirmed history of SARS-CoV-2 infection, based on at least one of the following criteria: *virological criteria *(detection of SARS-CoV-2 by RT-PCR from nasopharyngeal or oropharyngeal swabs, or protected distal sampling (PDP), as well as positive antigen or serological tests) and *radiological criteria *(patients classified as CO-RADS 5 on chest CT scans were considered COVID-19 positive, according to the COVID-19 Reporting and Data System (CO-RADS) classification).

Data were collected through patient interviews and review of medical records.

Ethical considerations

This study was approved by the University Hospital Ethics Committee of Tangier (Comité d'Éthique Hospitalo-Universitaire de Tanger (CEHUT)) (reference: Ac18fv/23). All patients were informed about the data collection process and were free to decline participation.

Variables and definitions

Demographic data collected included age, sex, and medical history such as hypertension, diabetes, cardiovascular disease, cancer, obesity (defined as BMI ≥30 kg/m²), smoking status, and the underlying nephropathy.

We also recorded vascular access characteristics at the time of COVID-19 diagnosis, access survival after infection, and the need for temporary access. Radiological evaluation included the level of COVID-19 suspicion based on the CO-RADS classification.

The extent of pulmonary involvement was assessed using a 0-5 scale based on the percentage of affected lung parenchyma: grade 1: <10% involvement, grade 2: 10-25%, grade 3: 25-50%, grade 4: 50-75%, and grade 5: >75%.

We recorded the need for hospitalization and the treatments received during the acute phase or post-COVID-19, including hydroxychloroquine, azithromycin, corticosteroids, anticoagulation, and oxygen therapy.

Persistent symptoms were evaluated at three months post-COVID-19 and included fatigue, weight loss, anorexia, dysgeusia, cough, dyspnea, arthralgia, sleep disturbances, concentration difficulties, and anxiety symptoms.

A favorable outcome was defined as the absence of persistent symptoms. An unfavorable outcome was defined by the presence of post-COVID-19 syndrome, also known as long COVID, which refers to prolonged symptoms that interfere with daily functioning and persist for at least two months after the initial infection [6].

Statistical analysis

Normality of data distribution was assessed using the Shapiro-Wilk and Kolmogorov-Smirnov tests. For normally distributed variables, results were expressed as means ± standard deviations. For non-normally distributed variables, medians and interquartile ranges (Q1-Q3) were reported. Categorical variables were described in terms of counts and percentages.

Comparisons between groups were performed using the Student’s t-test or Mann-Whitney U test for continuous variables, and the Chi-square test or Fisher’s exact test for categorical variables, as appropriate. Statistical significance was set at p < 0.05.

Odds ratios (ORs) with 95% confidence intervals (CIs) were calculated in bivariate analysis and included in a multivariate logistic regression model if p-values were <0.05. All statistical analyses were performed using IBM SPSS Statistics for Windows, Version 25.0 (released 2017, IBM Corp., Armonk, NY).

## Results

Over a 36-month period, among 945 patients on MHD, 163 (17.2%) were infected with SARS-CoV-2 and survived the acute phase of the illness. The overall clinical and demographic characteristics of the study population are detailed in Table 1. The median age of the infected patients was 55 years (IQR: 43-67), and the male-to-female ratio was 1.17. Diagnosis of COVID-19 was confirmed by a positive nasopharyngeal RT-PCR test in 49.6% of cases (n = 81). Regarding comorbidities, hypertension was the most common (40.5%, n = 66), followed by diabetes mellitus (32.5%, n = 53). Obesity (defined as BMI ≥ 30 kg/m²) was observed in 19% of cases (n = 31), and 11% of patients (n = 18) were active smokers. Before infection, 70.6% of patients (n = 115) had received a complete COVID-19 vaccination regimen.

**Table 1:** Characteristics of patients included in the study

Variables	N = 163
Age (years) median (Q1–Q3)	55 (43–67)
Sex ratio (M/F)	1.17
Dialysis vintage (years) median (Q1–Q3)	5.6 (3.1–8.7)
Hemoglobin (g/dL) mean ± SD	9.1 ± 1.5
Causal nephropathy: n (%)	
- Diabetes	50 (30.7%)
- Nephroangiosclerosis	28 (17.2%)
- Glomerulopathy	12 (7.4%)
- Interstitial	14 (8.6%)
- Uropathy	9 (5.5%)
- Hereditary	6 (3.7%)
- Undetermined	44 (26.9%)
COVID-19 disease characteristics	
- Extent of pneumonia (%) mean ± SD	91 ± 22
- Length of hospitalization (days) median (Q1–Q3)	15 (10–23)
- Duration of oxygen therapy (days) median (Q1–Q3)	21 (12.5–45)

Among the 163 infected patients, 11% required hospitalization (n = 18), and 10% of those hospitalized were admitted to an intensive care unit. The median length of hospitalization was 15 (IQR: 10-23) days.

At the time of diagnosis, an arteriovenous fistula (AVF) was the vascular access in 87.7% of patients (n = 143). AVF thrombosis occurred in 3.1% of patients (n = 5), and two of them required temporary vascular access placement.

From a respiratory standpoint, 12.3% (n = 20) of patients required oxygen therapy during the acute phase of the illness, and 5.5% (n = 9) required it beyond the acute period. The median duration of oxygen therapy was 21 (IQR: 12.5-45) days.

Twelve percent of patients (n = 20) received hydroxychloroquine, and azithromycin was prescribed in 80% of cases (n = 130). Corticosteroids were administered in 11.7% of cases (n = 19), and 11.04% of patients (n = 18) received anticoagulation therapy.

A favorable outcome was observed in 32% of cases (n = 52), while long COVID was reported in 68% of patients (n = 111), who presented with at least one persistent symptom two to three months post-infection. Among these, 29.4% experienced at least three persistent symptoms.

The most frequently reported post-COVID manifestations included fatigue (62%), anxiety symptoms (53%), arthralgia (40%), cough (33%), weight loss (29%), sleep disturbances (28%), anosmia (30%), dyspnea (25%), anorexia (24%), dysgeusia (18%), and concentration difficulties (16%). The median duration (in weeks) of the most common long COVID symptoms was anxiety symptoms in 11 (IQR: 8-17), fatigue in eight (IQR: 6-12), dyspnea in eight (IQR: 6-10), cough in seven (IQR: 7-9), and anosmia in seven (IQR: 6-8). The mean weight loss was 0.9 kg ± 1.40.

The frequency of long COVID was significantly higher (p < 0.05) among diabetic and obese patients, as well as those who required hospitalization or oxygen therapy, and those treated with hydroxychloroquine, corticosteroids, or anticoagulation. This likely reflects the increased severity of illness in these cases (Table 2).

**Table 2:** Comparison between patients with favorable outcomes and those with long COVID The data has been represented as N (%) or median (IQR). * Statistical significance was set at p < 0.05. ** Z score for Mann-Whitney U test (continuous variables) and chi-square value for categorical variables.

Variables	Favorable outcome (n = 52)	Long COVID (n = 111)	P-value	Test statistic numerical value **
			*	
Age median (Q1–Q3)	52 (39–69)	56 (44–66)	0.5	-0,625
Female sex n (%)	20 (38.5%)	55 (49.5%)	0.18	1.753
Dialysis vintage (years) median (Q1–Q3)	6.3 (3.8–8.7)	5 (2.7–8.9)	0.2	-1,05
Hypertension n (%)	17 (32.7%)	49 (44.1%)	0.16	1.927
Diabetes n (%)	7 (13.5%)	46 (41.4%)	0.0001	12.634
Obesity n (%)	4 (7.7%)	27 (24.3%)	0.012	6.36
Smoking n (%)	8 (15.4%)	10 (9%)	0.2	1.456
Vaccination n (%)	32 (61.5%)	83 (74.8%)	0.08	2.986
Oxygen therapy n (%)	1 (2%)	19 (17%)	0.006	7.594
Hospitalization n (%)	1 (2%)	17 (15.3%)	0.01	6.465
Hydroxychloroquine n (%)	1 (2%)	19 (17.1%)	0.006	7.594
Anticoagulation n (%)	1 (2%)	18 (16.2%)	0.008	7.025
Corticosteroid therapy n (%)	1 (2%)	19 (17.1%)	0.006	7.594

After adjusting for diabetes, obesity, the need for oxygen therapy or hospitalization, and the use of hydroxychloroquine or corticosteroids, the risk factor significantly associated with long COVID was diabetes (OR = 3.8; 95% CI (1.5-9.6); p = 0.004) (Table 3).

**Table 3:** Analysis of risk factors associated with long COVID

Risk factor	Univariate	95% CI	p-value	Multivariate	95% CI	p-value
Diabetes	4.5	1.9–11	0.001	3.8	[1.5–9.6]	0.004
Obesity	3.9	1.3–11.7	0.02	3	[0.9–9.7]	0.06
Oxygen therapy	10.5	1.4–81	0.02	1.2	[0.14–103]	0.9
Hospitalization	9.2	1.2–71	0.03	1.6	[0.03–87]	0.8
Hydroxychloroquine	10.5	1.3–81	0.02	2.7	[0.8–91]	0.5
Corticosteroid therapy	10.5	1.4–81	0.02	2.7	[0.8–91]	0.5

To refine our analysis of the most frequent long COVID symptoms, we investigated the risk factors associated with the persistence of specific symptoms beyond the acute phase of SARS-CoV-2 infection. 

After adjusting for diabetes, obesity, sex, the need for oxygen therapy or hospitalization, and the use of corticosteroids or hydroxychloroquine, the risk factors significantly associated with post-COVID fatigue (Table 4) were as follows: diabetes (OR = 2.9; 95% CI (1.3-6.5); p = 0.01), female sex (OR = 2.1; 95% CI (1.02-4.2); p = 0.04). Obesity approached statistical significance (OR = 2.9; 95% CI (0.9-9.7); p = 0.05).

**Table 4:** Risk factors associated with post-COVID fatigue (asthenia)

Risk factor	Univariate	95% CI	p-value
Diabetes	3.3	1.5–7.1	0.002
Obesity	3.9	1.4–10.9	0.008
Female sex	2	1.1–3.8	0.04
Hospitalization	5.6	1.2–25.5	0.03
Hydroxychloroquine	3.9	1.1–14.2	0.02
Corticosteroids	3.9	1.2–14.9	0.03
Oxygen therapy	6.5	1.5–29	0.01

After adjusting for diabetes, obesity, and the need for oxygen therapy or hospitalization, diabetes (OR = 2.6; 95% CI (1.2-5.5); p = 0.01), and obesity (OR = 2.5; 95% CI (1.01-6.4); p = 0.04) were associated with persistent anxiety symptoms (Table 5).

**Table 5:** Risk factors associated with persistent anxiety symptoms

Risk factor	Univariate	95% CI	p-value
Diabetes	3.3	1.6–6.6	0.001
Obesity	3.1	1.3–7.5	0.01
Hospitalization	8.6	1.9–38.6	0.005
Oxygen therapy	9.9	2.2–44.4	0.003

When adjusting for diabetes, obesity, hypertension, smoking status, oxygen therapy (and its duration), hospitalization, extent of lung lesions on chest CT, hemoglobin levels, and the use of corticosteroids and anticoagulation, only obesity (OR = 4.0; 95% CI (1.4-10.4); p = 0.004) was independently associated with persistent dyspnea (Table 6).

**Table 6:** Risk factors associated with persistent dyspnea

Risk Factor	Univariate	95% CI	p-value
Diabetes	2.2	1.1–4.6	0.03
Obesity	4.6	1.9–10.4	0.0001
Hypertension	2.7	1.3–5.5	0.007
Smoking	2.1	0.7–5.8	0.16
Duration of oxygen	1.7	0.9–3.0	0.07
CT lesion extent	1.13	1.01–1.2	0.03
Hemoglobin level	0.9	0.6–1.3	0.6
Oxygen therapy	18.8	5.8–61.2	0.000
Hospitalization	15.3	4.7–50.1	0.000
Corticosteroids	10	3.5–28.5	0.000
Anticoagulation	12.1	4–36.6	0.000

After adjusting for diabetes, obesity, smoking, hospitalization or oxygen therapy (and its duration), and the use of corticosteroids, anticoagulation, or hydroxychloroquine, diabetes was found to be significantly associated with persistent cough (OR = 2.5; 95% CI (1.2-5.2); p = 0.01) (Table 7).

**Table 7:** Risk factors associated with persistent cough

Risk factor	Univariate	95% CI	p-value
Diabetes	2.8	1.4–5.6	0.03
Obesity	1.9	0.9–4.2	0.12
Smoking	0.8	0.3–2.2	0.6
Duration of oxygen	1.04	0.9–1.1	0.16
Oxygen therapy	3.6	1.4–9.5	0.009
Hospitalization	3.7	1.4–10.3	0.01
Corticosteroids	2.3	0.8–5.8	0.09
Anticoagulation	2.5	0.9–6.6	0.06
Hydroxychloroquine	2.8	1.1–7.4	0.03

In a separate model with similar adjustments, obesity was also significantly associated with weight loss (OR = 3.0; 95% CI (1.2-7.3); p = 0.015) (Table 8).

**Table 8:** Risk factors associated with post-COVID weight loss

Risk factor	Univariate	95% CI	p-value
Diabetes	2.6	1.3–5.3	0.008
Obesity	3.3	1.5–7.5	0.004
Oxygen therapy	10	3.4–29.6	0.000
Hospitalization	8.1	2.7–24.5	0.000
Corticosteroids	7.5	2.7–20.9	0.000
Anticoagulation	6.8	2.4–19.1	0.000
Hydroxychloroquine	5.7	2.1–15.5	0.001

## Discussion

This study included 163 patients on MHD who had been infected with SARS-CoV-2. The median age of this population was 55.5 years (IQR: 43-67), consistent with national data on hemodialysis patients, where the median age is estimated at 53 years [7]. The male predominance observed in our cohort, along with the main etiologies for initiating dialysis, diabetes mellitus and hypertension, are also in line with epidemiological profiles reported in the literature [8]. A study by Savas Ozturk et al. involving a similar population reported a mean age of 64 years, a clear male predominance, and a high frequency of diabetic nephropathy and cardiovascular history [9].

MHD patients are at high risk of developing severe COVID-19 due to their immunosuppressed status, multiple comorbidities, and frequent exposure to healthcare settings [10].

In our study, nearly 75% (83/111) of patients who experienced long COVID had been fully vaccinated. A study supported by the UK’s National Institute for Health and Care Research (NIHR) showed that vaccination reduced the risk of developing long COVID symptoms by 12% after the first dose and by 21% after the second dose [11]. However, Sakhi et al. noted that although most dialysis patients develop a humoral response post-vaccination, the magnitude of this response is often lower compared to the general population [12].

Dialysis units represent high-risk environments for viral transmission. Frequent and close contact with healthcare personnel and other patients promotes rapid spread [13]. Even when seroconversion occurs post-infection or post-vaccination, the durability of the immune response remains uncertain. Several studies have demonstrated a significant decline in anti-SARS-CoV-2 IgG titers starting three months post-infection, potentially limiting protective immunity [14,15]. Current guidelines recommend administering three vaccine doses to MHD patients [16], including those previously infected, due to their attenuated immune response and the lack of strong consensus on post-infection immunity duration.

By contrast, vaccination was not significantly associated with the occurrence of long COVID in our cohort. This observation echoes the mixed results of other studies. Some research suggests a partial protective effect of vaccination, while others found no significant reduction in risk, particularly among patients with severe comorbidities such as those on dialysis [17]. It is possible that the impact of vaccination in our study was obscured by the severity of initial illness or limited statistical power due to sample size.

Since the onset of the pandemic, numerous studies have highlighted the persistence of symptoms several weeks or months after the acute phase of COVID-19. This condition, now referred to as long COVID, affects a significant proportion of patients, including those with mild initial infections [18]. Data on long COVID and persistent symptoms in MHD patients have expanded [19], although these symptoms appear similar to those reported in the general population [20]. The most common symptoms in our cohort three months after infection were fatigue (62%), anxiety (53%), arthralgia (40%), cough (33%), weight loss (29%), sleep disturbances (28%), and dyspnea (25%). These findings are consistent with those of Lopez-Leon et al., who reported over 50 persistent effects post-COVID, including fatigue (58%), headache (44%), attention disorders (27%), dyspnea (24%), anosmia (21%), and depressive or anxiety symptoms (up to 23%) [21].

Our study found that the prevalence of long COVID was significantly higher among diabetic, obese, hospitalized patients, those requiring oxygen therapy, and those treated with hydroxychloroquine, anticoagulants, or corticosteroids. This association with more severe initial clinical presentations suggests that the severity of acute COVID-19 may be a key determinant of persistent symptoms, consistent with previous research [22,23].

After multivariate adjustment, diabetes emerged as an independent risk factor for long COVID. This aligns with the literature, which highlights impaired immune response, chronic inflammation, and greater susceptibility to prolonged infections in diabetic patients [24]. Chronic hyperglycemia may also exacerbate endothelial dysfunction and prolong inflammatory responses.

Similarly, obesity was identified as a significant risk factor for post-COVID and persistent anxiety symptoms. This is supported by studies showing that obesity, as a chronic pro-inflammatory state, contributes to dysfunctional immune responses and prolongs post-infectious symptoms [25]. It has also been associated with slower recovery and poorer quality of life after SARS-CoV-2 infection [26].

Post-COVID fatigue was particularly common among diabetic and obese patients, likely due to a combination of metabolic, inflammatory, and psychological factors. Several cohorts, including that of Huang et al. [27], identified fatigue as the most common long-term symptom, especially in patients with underlying comorbidities [28].

Anxiety symptoms are frequently observed in post-COVID patients, reflecting the psychological impact of the acute illness, social restrictions, and prognostic uncertainty. A 2023 study by Montani et al. emphasized that psychiatric symptoms, including anxiety, are key components of the post-COVID syndrome [18]. Persistent dyspnea and sleep disturbances may further exacerbate psychological distress, creating a vicious cycle.

Weight loss may result from prolonged fatigue, gastrointestinal symptoms, or post-infectious anorexia. It is also an indirect marker of disease severity. Carriazo et al. (2021) reported that weight loss in post-COVID dialysis patients is a risk factor for prolonged morbidity [5], thus underlying the need for a personalized approach to address malnutrition.

Persistent dyspnea may indicate residual lung damage, muscle dysfunction, or dysregulated breathing. Huang et al. (2021) found dyspnea in up to 48% of patients six months post-infection [27], which aligns with our findings. Dyspnea is a major limiting factor and is often correlated with reduced functional capacity.

Routine screening for persistent symptoms in MHD patients could allow for early and targeted interventions, including tailored rehabilitation programs [29]. Some authors advocate for integrating post-COVID care into long-term dialysis follow-up and providing specialized rehabilitation to minimize functional decline and improve quality of life in this high-risk population [30].

Overall, our results highlight the need for individualized post-COVID care in chronic hemodialysis patients, especially those with diabetes or obesity. However, several limitations of our study should be acknowledged. First, important data such as albumin levels were unavailable for all patients, limiting a deeper analysis of nutritional or inflammatory factors. Second, the retrospective nature of the study introduces potential bias related to data collection and quality. Third, differences in patient management across centers may have influenced outcomes and limited the comparability of results. These factors should be considered when interpreting our conclusions.

## Conclusions

Our study highlighted a significant prevalence of long COVID among chronic hemodialysis patients, especially in patients with diabetes and obesity, thus underscoring the need for particular attention to this high-risk population and the necessity of routine screening for persistent symptoms after a SARS-CoV-2 infection in MHD patients.

Further studies are warranted to evaluate the potential protective effect of vaccination on the incidence of long COVID symptoms in chronic hemodialysis patients.

## References

[1] Thakur V, Ratho RK, Kumar P (2021). Multi-organ involvement in COVID- 19: beyond pulmonary manifestations. J Clin Med.

[2] Artborg A, Caldinelli A, Wijkström J (2024). Risk factors for COVID-19 hospitalization and mortality in patients with chronic kidney disease: a nationwide cohort study. Clin Kidney J.

[3] Bruchfeld A (2021). The COVID-19 pandemic: consequences for nephrology. Nat Rev Nephrol.

[4] do Nascimento Lima H, Nerbass FB, Neto OM, Sesso R, Lugon JR (2023). Chronic hemodialysis patients with COVID-19 cared for by the public health system have higher mortality than those treated in private facilities: analysis of the Brazilian dialysis registry. Int Urol Nephrol.

[5] Carriazo S, Mas-Fontao S, Seghers C (2022). Increased 1-year mortality in haemodialysis patients with COVID-19: a prospective, observational study. Clin Kidney J.

[6] (2021). World Health Organization. Post COVID-19 condition (long COVID). https://www.who.int/news-room/questions-and-answers/item/coronavirus-disease--post-covid-19-condition.

[7] Bahadi A, El Farouki MR, Zajjari Y, El Kabbaj D (2017). Hemodialysis in Morocco: the importance of nephrological monitoring [Article in French]. Nephrol Ther.

[8] Bulut C, Kato Y (2020). Epidemiology of COVID-19. Infect Dis Clin Microbiol.

[9] Sallustio F, Curci C, Chaoul N (2021). High levels of gut-homing immunoglobulin A+ B lymphocytes support the pathogenic role of intestinal mucosal hyperresponsiveness in immunoglobulin A nephropathy patients. Nephrol Dial Transplant.

[10] Araeipour-Tehrani Y, Haidar F, Saudan P (2021). Impact of the COVID-19 pandemic on patients with chronic kidney disease. Rev Med Suisse.

[11] Martí Català N, Mercadé-Besora N, Kolde R (2024). Effectiveness of COVID-19 vaccines to prevent long COVID symptoms: staggered cohort study from the UK, Spain, and Estonia. Lancet Respir Med.

[12] Lano G, Sallée M, Pelletier M (2022). Neutrophil:lymphocyte ratio correlates with the uremic toxin indoxyl sulfate and predicts the risk of death in patients on hemodialysis. Nephrol Dial Transplant.

[13] Kliger AS, Silberzweig J (2020). Mitigating risk of COVID-19 in dialysis facilities. Clin J Am Soc Nephrol.

[14] Earle KA, Ambrosino DM, Fiore-Gartland A (2021). Evidence for antibody as a protective correlate for COVID-19 vaccines. Vaccine.

[15] Seow J, Graham C, Merrick B (2020). Longitudinal observation and decline of neutralizing antibody responses in the three months following SARS-CoV-2 infection in humans. Nat Microbiol.

[16] Agur T, Ben-Dor N, Goldman S (2022). Immunogenicity of a third dose of BNT162b2 COVID-19 vaccine in maintenance hemodialysis patients. Nephrol Dial Transplant.

[17] Antonelli M, Penfold RS, Merino J (2022). Risk factors and disease profile of post-vaccination SARS-CoV-2 infection in UK users of the COVID Symptom Study app: a prospective, community-based, nested, case-control study. Lancet Infect Dis.

[18] Montani D, Savale L, Noel N (2023). Post-COVID-19 syndrome. Bull Acad Natl Med.

[19] Cheng Y, Luo R, Wang K (2020). Kidney disease is associated with in-hospital death of patients with COVID-19. Kidney Int.

[20] Lazzez R, Toumi S, Ben Achour N (2021). Post-COVID in chronic hemodialysis [Article in French]. Nephrol Ther.

[21] Lopez-Leon S, Wegman-Ostrosky T, Perelman C, Sepulveda R, Rebolledo PA, Cuapio A, Villapol S (2021). More than 50 long-term effects of COVID-19: a systematic review and meta-analysis. Sci Rep.

[22] Nalbandian A, Sehgal K, Gupta A (2021). Post-acute COVID-19 syndrome. Nat Med.

[23] Carfì A, Bernabei R, Landi F (2020). Persistent symptoms in patients after acute COVID-19. JAMA.

[24] Apicella M, Campopiano MC, Mantuano M, Mazoni L, Coppelli A, Del Prato S (2020). COVID-19 in people with diabetes: understanding the reasons for worse outcomes. Lancet Diabetes Endocrinol.

[25] Caussy C (2021). Obesity and COVID-19 infection: a dangerous liaison [Article in French]. Med Mal Metab.

[26] Garrigues E, Janvier P, Kherabi Y (2020). Post-discharge persistent symptoms and health-related quality of life after hospitalization for COVID-19. J Infect.

[27] Huang C, Huang L, Wang Y (2021). 6-month consequences of COVID-19 in patients discharged from hospital: a cohort study. Lancet.

[28] Sandler CX, Wyller VB, Moss-Morris R (2021). Long COVID and post-infectious fatigue syndrome: a review. Open Forum Infect Dis.

[29] Bileviciute-Ljungar I, Schult ML, Borg K, Ekholm J (2020). Preliminary ICF core set for patients with myalgic encephalomyelitis/chronic fatigue syndrome in rehabilitation medicine. J Rehabil Med.

[30] Chawki S, Buchard A, Sakhi H (2022). Long-term impact of COVID-19 among maintenance haemodialysis patients. Clin Kidney J.

